# Inclusive Education, Intellectual Disabilities and the Demise of Full Inclusion

**DOI:** 10.3390/jintelligence12020020

**Published:** 2024-02-11

**Authors:** Garry Hornby, James M. Kauffman

**Affiliations:** 1 Institute of Education, University of Plymouth, Plymouth PL4 8AA, UK; 2 School of Education and Human Development, Department of Special Education and Disability, University of Virginia, Charlottesville, VA 22908, USA; jmk9t@eservices.virginia.edu

**Keywords:** intellectual disabilities, full inclusion, inclusive education, special education

## Abstract

Inclusive education has developed worldwide popularity in education for learners with various disabilities but is particularly controversial for students with intellectual disabilities because of their unique needs. The foremost of these are the development of the social, vocational and life skills needed to facilitate their transition to adulthood. This article presents a discussion that focuses on theory, practice and research relevant to inclusive education for students with intellectual disabilities. It points out that the movement for full inclusion started by focusing on students with intellectual disabilities and has encountered roadblocks to further progress because of its difficulties in addressing their special needs. This is explored by considering the theory underpinning the international drivers of the full inclusion movement, the reality of the implementation of inclusion policies worldwide, and research on the effects of inclusion of students with intellectual disabilities in mainstream schools.

## 1. Introduction

The beginnings of special education can be traced back to the late eighteenth and early nineteenth centuries in Europe. Then, in the early to mid-nineteenth century, special schools for children who were blind or deaf and those with severe intellectual or other mental disabilities were established in the United States (Hallahan et al. 2023). By the mid-nineteenth century, most Western countries were trying to ensure that all children attended school at the elementary (or primary) school level. By the late nineteenth century, this resulted in teachers attempting to cope with a much wider range of students’ intellectual abilities than previously. Educators and legislators found that many children were struggling to develop basic academic skills, and this was recognized in all nations with mandatory universal education.

In France, in 1905, Alfred Binet developed a test to identify children with difficulties in learning so that they could be given remedial assistance. In the United States and Europe, over the next two decades, an adaptation of Binet’s test led to the development of what has become known as intelligence tests that can identify children with intellectual disabilities severe enough to make it difficult for them to learn effectively in mainstream classrooms. Binet’s intelligence tests were used to derive an “intelligence quotient” or IQ, so such tests of intelligence came to be known as “IQ tests”.

Throughout the first half of the 20th century, these IQ tests were used to select children for placement in special schools and special classes within mainstream schools, which led to steady growth in the number of special schools and classes for children with moderate and severe intellectual disabilities that continued in subsequent decades (Kauffman and Gerber, forthcoming; Kode 2017).

However, all tests used to measure intelligence result in a continuous statistical distribution, and the best cut point on that distribution for identifying an intellectual disability, as well as the instruments themselves, have been controversial for many decades (Kauffman and Lloyd, forthcoming). Also, findings of disproportional representation of minorities raised issues of cultural bias in testing and identification.

In 1968, Lloyd Dunn published a landmark article claiming that some children in the United States had been wrongfully placed in special classes for pupils with intellectual disabilities and were receiving a substandard education, so should not be legitimately considered “mentally retarded” (the term used in that era for what is today called intellectual disability). Dunn (1968) was referring to children with mild intellectual disabilities (those with IQs 70 to 85, one or two standard deviations below the population mean). He argued that these pupils, most of whom were in classes he observed, were being denied equal opportunities for development because they were placed in separate (“segregated”) special classes taught exclusively by special education teachers. This, he suggested, was unjustifiable because their intellectual disabilities were only mild, or in fact non-existent because of racial or cultural biases in testing and identification procedures. The solution to this issue, he suggested, was to place such children in mainstream classrooms with special educators acting as resource teachers, rather than children being educated full time in special classes.

However, Dunn’s (1968) article was somewhat ambiguous, by his own admission (Dunn 1983). The question then and now is where to draw the line—what IQ and other criteria to use in defining mental retardation or intellectual disability. In any case, separate special education for students with intellectual disabilities had been called into question by an influential leader in special education.

Unfortunately, in our opinion, the ambiguity of Dunn’s article led some educators to question the existence of *all* separate special classes for children with *all* types of disabilities at *all* levels. This eventually led to the full inclusion movement (Fuchs and Fuchs 1994). This movement, supporting the inclusion of *all* children with special educational needs or disabilities, has now become a controversial issue worldwide (Ahrbeck et al. 2018; Anastasiou et al. 2018, 2024; Gordon-Gould and Hornby 2023; Hornby and Kauffman 2023; Kauffman et al. 2022a, 2023a, 2023b).

The *Education for All Handicapped Children Act of 1975* in the USA (EAHCA 1975, also called Public Law 94-142) was landmark legislation in the field of special education internationally (Martin 2013). EAHCA was renamed the *Individuals with Disabilities Education Act* in 1990 and was reauthorized in 2004. It remains extremely influential in the United States and many other countries around the world. IDEA (2004) specifies the education entitlements of children with disabilities and promotes their education within the “least restrictive environment” (LRE). Focus on the LRE is considered by some people to have encouraged the movement toward the inclusion of learners with all types of disabilities within mainstream, regular, or general education classrooms in various countries across the world.

Concern about the LRE is considered to have overshadowed other more important aspects of the United States federal law (Hallahan 2024; Kauffman et al. 2022c), as well as special education policies throughout the world (Gordon-Gould and Hornby 2023; Hornby and Kauffman 2023; Kauffman et al. 2022a; Slee 2018a, 2018b). Under U.S. federal law, each child with a disability is to have an individual education program (an IEP), and that IEP is to be implemented in the LRE chosen from a continuum of alternative placements (CAP). As Bateman and Linden (2012) and others have pointed out, the LRE is considered last, cannot be determined before the IEP is written, and must be chosen from the CAP. The US law refers to a continuum of *placements*, not services, but a variety of services is implied by other provisions of the law (Yell and Prince 2022; Yell and Bradley 2024).

So, it appears that it was essentially dissatisfaction with the quality of education in special classes for students with intellectual disabilities in the United States that first led to questioning the appropriateness and equitability of ‘segregated’ special education for children with disabilities worldwide. However, it is considered by many special education teachers that instruction, not placement, should be the primary concern of educators (Hornby 2021; Hornby and Kauffman 2023; Kauffman and Badar 2014, 2016, 2020; Kauffman et al. 2020). Furthermore, the apparent solution to the issue of inequity was seen by many to be like other issues of diversity, one of inclusion of all children with disabilities, regardless of type or degree, in mainstream educational settings. This eventually led to a consideration of whether special classes and special schools were a necessary component of education systems in order to ensure equity and optimize educational outcomes for all children (Slee 2018a, 2018b).

Osgood (2005) noted that, although many children with disabilities moved into mainstream schools, many of those with intellectual disabilities continued to be separated into special education classes. For example, despite the Regular Education Initiative (see Lloyd et al. 1991) in the United States promoting the development of inclusive practices in mainstream schools to meet the needs of all students, there has been a continued growth of separate special education arrangements. This may be related to the increasing number of charter schools in the USA and academies in the UK that enroll few if any students with intellectual disabilities because of their focus on raising academic attainment levels and reducing costs, which poses a threat to inclusive practices (Dudley-Marling and Baker 2012; Gordon-Gould and Hornby 2023). Therefore, it is notable that most students with severe and profound intellectual disabilities continue to be placed in special schools (see Agran et al. 2020; Kauffman et al. 2020).

## 2. Roadblocks to the Progress of Inclusion for Students with Intellectual Disabilities

### 2.1. Theory Driving Inclusion Policies

The first roadblock to the progress of inclusion for students with intellectual disabilities is inconsistencies in the theory underpinning the international drivers of the inclusion movement, a major aspect of which is the Salamanca Statement and Framework for Action on Special Needs Education (UNESCO 1994). The statement and framework were outcomes of a conference addressing international concerns for about 350 million children globally who were at that time excluded from their nations’ education systems. This exclusion of children from education was highlighted by the World Conference on Education for All conference that was held in Jomtien, Thailand (UNESCO 1990). The focus of conference attendees was to universalize primary education and massively reduce illiteracy by the end of the decade.

The Salamanca Statement and Framework for Action on Special Needs Education (UNESCO 1994) was focused on enabling as many children with disabilities as possible to be included in nations’ regular education systems. It stated that regular schools with an inclusive orientation can “…provide an effective education for the *majority* of children” (our italics) and concluded that “ … special schools or units within inclusive schools may continue to provide the most suitable education for the relatively small number of children with disabilities who cannot be adequately served in regular classrooms or schools (UNESCO 1994, section 9)”.

It is clear that the Salamanca Statement considered that all children with disabilities should be included within nations’ education systems, but recognized that, although the majority of them could be included in mainstream schools, a minority would need to be educated in separate special schools or classes within mainstream schools.

Therefore, the Salamanca statement did *not* support the idea of full inclusion, with all children with disabilities being educated in mainstream classrooms and schools. Unfortunately, many authors have referred to the Salamanca statement, explicitly or by inference, as if it did recommend *full* inclusion (e.g., Ainscow 2020; Ainscow et al. 2019; Slee 2018a, 2018b). This is clearly a misrepresentation, but it has been repeated so many times that it has come to be thought by many to be a fact, often supported by the statement, “all means all” (see Sailor et al. 2021; Slee 2018b; SWIFT Schools 2023). Therefore, many educators and parents have been led to believe that full inclusion was recommended by the Salamanca Statement, although this is clearly not true. Many people have believed this myth, which has been repeatedly disseminated through journal articles, books, websites, and official documents. It has contributed to the myth that full inclusion is a realistic aim for the education of *all* children with disabilities, including those with moderate, severe and profound intellectual disabilities (Hornby and Kauffman 2023).

One consequence of this myth is the promotion of the policy of full inclusion by General Comment Number Four of Article 24 of the United Nations Convention on the Rights of Persons with Disabilities (UNCRPD 2016), which effectively proposed the end of special education, as it called for all countries to implement fully inclusive education systems as soon as possible. This was partly based on the controversial statement that children with disabilities educated in separate settings receive an education of inferior quality, which has been strongly disputed by many special educators (Anastasiou et al. 2018; Fuchs et al. 2023; Gordon-Gould and Hornby 2023; Hyatt and Hornby 2017; Kauffman 2020, 2022, forthcoming). Given this unsubstantiated claim, important questions about the implications of moving to and operating fully inclusive education systems have been raised, as have questions about the future of special education. Scrutiny of this is necessary because the policy directive of UNCRPD Article 24 foreshadows the extinction of special education, possibly along with the loss of decades of development of expertise, techniques, and technology for effectively educating children with disabilities.

### 2.2. The Practice of Inclusion

The second roadblock to the progress of inclusion for students with intellectual disabilities is the claim that full inclusion is feasible because it has existed in Italy since its national government closed all special schools in 1977. This is used as an example by those who believe that full inclusion of all children with disabilities, with no exceptions, is a viable option (e.g., Ainscow 2020; Begeny and Martens 2007). However, an examination of the education system in Italy shows that it is not an example of the viability of full inclusion. Actually, since 1977, Italy has maintained public funding for special schools. In 2011, there were reported to be 71 separate special schools in Italy (EADSNE 2012). Anastasiou et al. (2015) concluded that

Despite the promises of full inclusion, the everyday reality in Italian classrooms is more complex… it seems that an informal “backdoor” special education has been constructed and developed by schools at the local level to address specialized educational needs.

(p. 439)

More recently, the feasibility of full inclusion has been promoted by citing the education system in the Canadian province of New Brunswick as an example of the success of full inclusion. For example, “In the last three decades, this small Canadian province has implemented a model of inclusive schooling for all students, including those previously served by special education programs” (AuCoin et al. 2020, p. 314). However, despite the fact that New Brunswick’s full inclusion model was implemented over three decades ago, there are no published studies evaluating its effectiveness in educating children with disabilities or comparing such outcomes with other Canadian provinces that have maintained special schools and special classes.

Furthermore, a Google search turns up many newspaper articles reporting teachers and parents in New Brunswick complaining that children with disabilities are being denied an effective education because of the full inclusion model (e.g., CBC News 2017; Doherty 2012). There are also articles critical of the New Brunswick full inclusion model by local education experts (e.g., Bennett 2017) and by the New Brunswick Education Minister, who has called for a review of the inclusion policy, because it, “… isn’t working effectively for students with developmental disorders, other students in the classroom and teachers” (CBC News 2020). It appears that the complexity of implementing a full inclusion model has not been as simple as it sounds (Kauffman et al. 2022d).

Realizing that, according to Imray and Colley (2017), achieving full inclusion has not been feasible in any country, ought to be making policy makers extremely wary about attempting to implement fully inclusive education systems where young people with intellectual disabilities are educated in mainstream schools.

In practice, one must draw lines indicating criteria that define a disability, and this applies to all categories of exceptionality. Drawing the line defining intellectual disabilities was highlighted by Dunn (1968) decades ago. The problems of drawing lines defining all categories of disabilities, intellectual disabilities included, are intractable–irresolvable except by criteria that are based on the best estimates we can make of the risks and benefits of drawing the line at a particular value (of IQ, adaptive behavior, etc.; Kauffman and Lloyd, forthcoming; Kauffman et al. 2022b). Unsurprisingly, one response (popularized by the field of disability studies in education) is to deny that categories of disability exist, and instead consider all disabilities normal variations or diversities. The consequences of this view are disastrous for people with disabilities since the impairments associated with intellectual disabilities are real and not social constructions (Hallahan 2024).

The practicalities and challenges involved in implementing inclusion have been clearly described by Robbins (2023). She reports that many general education teachers are willing to work with special education students, including many of those with intellectual disabilities, and that inclusion sometimes does work well. However, suggesting that it is *always* the best choice or is *always* feasible appears to be a fantasy not consistent with the realities of schools. In remarking on the role of imagination in reforming or rethinking education, Kauffman et al. (2022a) noted that

Our intention is to approach imagination of the future of special and inclusive education with open minds. However, we also realize that this means our minds are not open to each and every possible imaginary future. We believe, like physicist Lawrence Krauss, that “A truly open mind means forcing our imaginations to conform to the evidence of reality, and not vice versa, whether or not we like the implication”.

(Krauss 2012, p. 140)

### 2.3. Research on the Effectiveness of Inclusion

The third roadblock to the progress of inclusion for students with intellectual disabilities is the lack of research evidence for the effectiveness of inclusion, that is, demonstrating that it is more effective than separate special education interventions. Research to date does not support the claims that inclusion has been shown to be more effective (e.g., Anastasiou et al. 2015; Cook and Cook 2020; Fuchs et al. 2023; Hornby and Kauffman 2023; Imray and Colley 2017). However, because it is unethical to conduct randomized control trials of full inclusion versus separate special education, making a definitive statement about the superiority of either of these for *all* students is just not possible. But this has not stopped some writers from claiming that there is a solid research evidence base for the effectiveness of inclusion (e.g., De Bruin 2020; SWIFT Schools 2023). Such claims usually cite studies and reviews of studies with serious flaws in research methodology, which makes inferences from them invalid (e.g., Hehir 2016).

In fact, recent reviews of the literature have concluded that there is no consistent evidence for the advantage of either special education or inclusive education (Dalgaard et al. 2022; Stephenson and Ganguly 2021). However, a major source of evidence for the effectiveness of interventions in the field of education, Hattie’s (2009) synthesis of meta-analyses, has reported that the effect size for inclusive education interventions is 0.25. This is well below the average of 0.4 found by Hattie for educational interventions overall, suggesting that inclusive education interventions have a low level of effectiveness.

Over the past 20 years, several studies have found that special education interventions can be effective in improving the outcomes of young people with disabilities. For example, Hanushek et al. (2002) used repeated performance measures to identify program effects by comparing the achievement gains of students who spent time in both special education and regular education. They found that, on average, special education programs had significantly beneficial effects on performance, and that this was particularly true for students with learning disabilities. Likewise, Hurwitz et al. (2019) compared students’ performance on standardized tests before, during, and after special education placement and found that students’ scores not only improved but showed a sustained trajectory of academic growth following their involvement in special education. Further, Schwartz et al. (2019) found that academic outcomes for students with learning disabilities improved following their entry into special education and that the impacts were largest for those who entered during earlier grades.

Clearly, more research is needed to investigate the comparative effectiveness of special education and inclusive education. Moreover, a more accurate interpretation of the existing research literature is an essential part of attempts to avoid confusion and misrepresentation, as Oreskes (2023) indicated in a recent essay about the confusion of scientific evidence in which she showed how experimental rigor can cloud reality, concluding that scientific questions and methodology must fit the issue at hand (see also Gloski et al. 2022). The key issue regarding special education is the effectiveness of interventions, including placement, on student achievement. This is why studies of long-term outcomes for students with intellectual disabilities are particularly important.

## 3. Research on Long-Term Outcomes for Students with Intellectual Disabilities

Long-term outcomes, such as examining the extent to which young people with intellectual disabilities are included in their communities after leaving school, are arguably the most important measure of concern because they evaluate the extent to which the principal educational goal—inclusion in the community—has been achieved. These studies are difficult to conduct because of the extended times involved so are rare in the literature. However, two studies that one of the authors has been involved with provide some evidence about the outcomes of special education and inclusion even though they are now somewhat dated. These studies involved young people with intellectual disabilities, referred to as having moderate learning difficulties, typically considered to have IQs ranging from 50 to 75. The first is an anecdotal report of a case study of teenagers with moderate learning difficulties taught in a secondary school special class in New Zealand, who left school from the special class in the late 1970s. The second is a follow-up study of young people with moderate learning difficulties in the north of England in the 1990s. In this study, participants were included in mainstream schools for the last few years of their schooling, after being in a special school for most of their school lives.

### 3.1. Case Study of Graduates of a Special Class in New Zealand

For three years, between 1974 and 1976, author Hornby taught young people with moderate learning difficulties aged 14 to 16 years in a special class within a mainstream secondary school in New Zealand. A social and vocational training curriculum was used, with those in the second year of the two-year program spending one day per week in “work experience” jobs, organized and supervised by the class teacher. The focus of work in the classroom was on functional academics, daily living skills, social skills and vocational training. They did not follow the academically focused New Zealand National Curriculum, but instead followed a curriculum designed to match the needs of the students. Special class activities included: class discussion, problem-solving and role play of challenging situations; functional reading such as completing application forms and finding information from newspapers; using listening posts for learning the New Zealand Road Code; work simulations using an in-class production line; shopping for ingredients and cooking lunch in groups of three; playing table tennis in the classroom for social skill development; trips to the city to observe people at work, attend film theatres and gain independence; and, work experience one day per week in the second year to develop vocational skills.

Following their time in the special class, the vast majority of young people who left school secured jobs in open employment and only two were unable to do this, so they attended sheltered workshops. Many were employed in one of the jobs they worked for one day per week of their work experience. Some informal follow-up work was carried out about three years after they had left school and it was found that the special class graduates typically had kept their jobs or had obtained new ones. Few were unemployed and several owned their own cars. These anecdotal findings supported the view that for young people with moderate learning difficulties a vocational curriculum, including work experience, in their last few years at school, helped them gain employment and have a satisfactory quality of life. Similar findings have been reported in international studies and reviews of research on this issue, supporting the conclusion that vocational curricula and work experience are key factors in achieving positive outcomes for young people with moderate learning difficulties (Blackorby and Wagner 1996; Malkani 2021; Phelps and Hanley-Maxwell 1997).

### 3.2. Follow-Up Study of Young People with Moderate Learning Difficulties in England

A study was conducted by author Hornby with an ex-principal of a special school, who had been employed to close down a special school for students with moderate learning difficulties in the north of England by including all the young people in mainstream schools for the last few years of their school careers (Kidd and Hornby 1993). Twenty-nine students were transferred from a special school for young people with moderate learning difficulties to mainstream schools, with teacher-aide support organized by the ex-principal. The first part of the study involved interviews conducted by the ex-principal with the young people and their parents soon after their transfer to mainstream schools (Kidd and Hornby 1993). These were followed up by more interviews several years later, once all the young people had finished school, and they were at an average age of 22 years (Hornby and Kidd 2001). At this later time, out of the 29 young people who were transferred to mainstream schools, 24 were located and interviewed. They had spent an average of seven years at the special school and three years in mainstream schools following this to complete their schooling. Most the young people had followed a mainly academic curriculum in their mainstream schools, although some were placed in a special class and some of these were able to take part in work experience. Eleven out of the 12 who had been in a special class within a mainstream school viewed their transfer positively, compared with only 4 out of the 12 who were transferred into mainstream classes, while 8 saw it negatively. This difference was also found in their parents’ views, with more parents of children transferred to the special class satisfied with this placement than those whose children had been placed in mainstream classrooms.

In the second round of interviews, conducted when the young people were an average age of 22 years, 17 out of the 24 young people were found to be unemployed, and only three were working full time (Hornby and Kidd 2001). Eight out of the nine who had held jobs at some stage after leaving school had had work experience at secondary school, or Further Education College, compared with only 4 out of the 15 who had not had any work experience. Out of the 24 interviewed, 17 were living with their parents, while only 4 were living independently of their parents. Sixteen out of the 24 were receiving severe disability allowance, which meant they were deemed unable to be employed, so there were no attempts being made to find them jobs. This shocked the ex-principal of the special school who had organized their transfer to mainstream schools, as he considered that most of them should have been able to work in open employment given what he knew about them. The outcomes were therefore considered very poor and were extremely alarming to the former principal as it appears that the inclusion of these young people with intellectual disabilities in mainstream schools for the last few years of their school lives had been counterproductive.

Comparing the outcomes from the English and New Zealand studies, it is clear that outcomes were much better for those young people with intellectual disabilities who left school directly from a special education class than those who were included in mainstream schools for the last few years of their school lives. The findings of these long-term outcome studies conducted in two countries with very different policies on inclusion have implications for the education of young people with various levels of intellectual disabilities in many countries around the world.

## 4. Conclusions

The movement for full inclusion started by focusing on the education of students with intellectual disabilities but has encountered roadblocks to the implementation of full inclusion because of the practical difficulties of addressing the unique needs of students with intellectual disabilities in mainstream schools and the accumulating evidence that special education placements appear more effective in achieving better outcomes in terms of their inclusion in their communities post school.

We therefore conclude that, after many years of unsuccessful attempts to establish an idealistic vision of full inclusion for students with disabilities in education systems in different parts of the world, the full inclusion movement has come to a dead-end because of its inability to demonstrate effective inclusion of students with intellectual disabilities. This has come about through the realization of its inability to overcome the difficulties of providing many students with intellectual disabilities with an appropriate and effective education in mainstream classrooms in a full-inclusion education system, which has resulted in their continued placement in special classes and special schools in many countries around the world.

It is clear from the evidence that we have presented that continuing to promote a full inclusion agenda is likely to diminish effective education for many young people with intellectual disabilities. It is therefore essential that supporters of full inclusion recognize the real risks of continuing to promote such an unrealistic agenda and recognize the need to follow a more pragmatic path in the future for young people with intellectual disabilities. Therefore, in the absence of contradictory evidence, it is considered important that organizations such as UNESCO (2020) and the European Agency for Special Needs and Inclusive Education (EASNIE 2022), which both promote full inclusion, re-evaluate their positions in order to adopt more realistic policies, especially for young people with intellectual disabilities. Such policies should integrate what has been learned about inclusive education and special education in order to promote approaches that are more likely to lead to the implementation of effective education systems for optimizing outcomes for all young people with special educational needs and disabilities, including those with intellectual disabilities.

Rather than having either inclusion or special education, we consider that *both* inclusive education *and* special education are necessary to achieve optimal outcomes for students with intellectual disabilities. What we propose is that education systems implement policies that combine the philosophy and values of inclusive education with strategies and programs from special education so they are both equitable and effective for all learners, including those with intellectual disabilities. This approach has been referred to as Inclusive Special Education (Hornby 2014, 2015) and involves a recognition that all children with disabilities can be provided for appropriately within education systems that combine effective mainstream schools with high-quality special education provision. It considers that, although most children can be educated in mainstream classrooms, a relatively small number (such as many of those with moderate, severe and profound intellectual disabilities) benefit more by being taught in special classes within mainstream schools or being educated at special schools, whether they are on the campuses of mainstream schools or their own sites. Mainstream schools need to work closely with special schools to enhance their provision of support for milder degrees of special educational needs and disabilities while special schools provide education for children with more severe levels of disability (Hornby 2014, 2015). When Inclusive Special Education is implemented, mainstream schools are organized to provide effectively for a wide range of children with special needs by using programs and strategies that are evidence-based and have been found to be the best practices for supporting the education of all learners, including those with intellectual disabilities (Hornby and Greaves 2022).

Inclusive Special Education requires a commitment to providing effective education for all children with disabilities, in the most appropriate setting for the individual, throughout all stages of education. Its focus is on the inclusion of children with special educational needs and disabilities in mainstream schools as far as it is appropriate, along with the availability of a continuum of placement options from mainstream classes to special schools. It involves the ongoing close collaboration between teachers in mainstream and special schools and classes, in order to ensure equitable quality of appropriate provision and optimum outcomes for all learners.

## Data Availability

Data referred to in this article are available from the sources that are cited.
